# Resistance to Some New Drugs and Prevalence of ESBL- and MBL-Producing Enterobacteriaceae Uropathogens Isolated from Diabetic Patients

**DOI:** 10.3390/life12122125

**Published:** 2022-12-16

**Authors:** Othman M. Alzahrani, Fakhur Uddin, Samy F. Mahmoud, Amal S. Alswat, Muhammad Sohail, Mona Youssef

**Affiliations:** 1 Department of Biology, College of Science, Taif University, P.O. Box 11099, Taif 21944, Saudi Arabia; 2 Medical Technologist, Department of Microbiology, Basic Medical Sciences Institute (BMSI), JPMC, Karachi 75510, Pakistan; 3 Department of Biotechnology, College of Science, Taif University, P.O. Box 11099, Taif 21944, Saudi Arabia; 4 Department of Microbiology, University of Karachi, Karachi 75270, Pakistan; 5 Department of Hepatology, Gastroenterology and Infectious Diseases, Benha Teaching Hospital, Benha 13518, Egypt

**Keywords:** antimicrobial resistance, β-lactamase, *E. coli*, *K. pneumoniae*, type 2 diabetes

## Abstract

Diabetes is a leading non-communicable disease and a risk factor for relapsing infections. The current study was aimed at investigating the prevalence and antibiotic susceptibility of carbapenem-resistant (CR) uropathogens of the family Enterobacteriaceae in diabetic patients. The data of 910 bacterial isolates was collected from diagnostic laboratories during January 2018 to December 2018. The bacterial isolates were identified using traditional methods including colonial characteristics, biochemical tests, and API (20E). Antimicrobial susceptibility and phenotypic characterization of ESBL, MBLs, and KPC was determined by utilizing CLSI recommended methods. The phenotypically positive isolates were further analyzed for resistance-encoding genes by manual PCR and Check-MDR CT103XL microarray. Susceptibility to colistin and cefiderocol was tested in accordance with CLSI guidelines. The data revealed that most of the patients were suffering from type 2 diabetes for a duration of more than a year and with uncontrolled blood sugar levels. *Escherichia coli* and *Klebsiella pneumoniae* were the most frequently encountered pathogens, followed by *Enterobacter cloacae* and *Proteus mirabilis*. More than 50% of the isolates showed resistance to 22 antibiotics, with the highest resistance (>80%) against tetracycline, ampicillin, and cefazolin. The uropathogens showed less resistance to non-β-lactam antibiotics, including amikacin, fosfomycin, and nitrofurantoin. In the phenotypic assays, 495 (54.3%) isolates were found to be ESBL producers, while ESBL-TEM and -PER were the most prevalent ESBL types. The resistance to carbapenems was slightly less (250; 27.5%) than ESBL producers, yet more common amongst *E. coli* isolates. MBL production was a common feature in carbapenem-resistant isolates (71.2%); genotypic characterization also validated this trend. The isolates were found to be sensitive against the new drugs, cefiderocol and eravacycline. with 7–28% resistance, except for *P. mirabilis* which had 100% resistance against eravacycline. This study concludes that a few types of ESBL and carbapenemases are common in the uropathogens isolated from the diabetic patients, and antibiotic stewardship programs need to be revisited, particularly to cure UTIs in diabetic patients.

## 1. Introduction

Diabetes mellitus is one of the leading non-communicable diseases (NCD), ranked at number 5 among NCDs at a global level [1,2]. For the past 3–4 decades, a surge in diabetic cases has been witnessed. Due to a less established health care sector, the situation is worse in developing countries, and hence, a large population remains at risk of complications caused by diabetes. An unregulated and elevated level of blood glucose incurs damages to multiple organs, including the blood vessels, retinas, and kidneys, leading to various complications [3,4]. Neuropathy-related incomplete emptying of the bladder, poor metabolic control, and a weak immune system are amongst the risk factors causing repeated UTIs in diabetic patients [5]. The burden of UTIs and other complications in diabetic patients keep health care facilities under pressure, even in developed countries [6,7,8]. It is indeed reported that UTIs in diabetic patients exhibit more severe symptoms such as emphysematous cystitis, pyelonephritis, renal abscesses, and papillary necrosis [5] and result in longer hospital stays. Deplorably, the management of UTIs in diabetic patients is mainly concomitant with poor outcomes as compared to non-diabetic patients [9].

Bacteria commonly encountered in UTIs [10] include *Escherichia coli* and *Klebsiella pneumoniae* along with other Enterobacteriaceae members [11,12,13]. Indeed, diabetic patients are mainly infected by highly resistant pathogens, including extended-spectrum β-lactamase (ESBL)-producing Enterobacteriaceae and carbapenem-resistant Enterobacteriaceae [5,14]. β-lactam antibiotics are frequently prescribed drugs because of their broad spectrum and safety profile [10,15,16]. Different resistance mechanisms against β-lactams include alterations in the drug target site, decreased membrane permeability, and the activity of the drug efflux pump, but β-lactamases, especially extended spectrum β-lactamases (ESBLs) and carbapenemases [16], are the most common features. ESBL producers are the leading cause of higher morbidity and mortality in UTI patients [17]. This may be attributed to the overuse of antibiotics or to the ability of the microbes to grow as biofilms, or caused by genetic means, including mutations and the horizontal transfer of resistance genes [11].

Several studies describe specific genotypes in ESBL-producing pathogens causing resistance to some antibiotics [17,18]. In India, TEM (temoniera) has frequently been reported among uropathogens [17]. Whilst in Europe, United States, and Africa, CTX-M (cefotaximase) has remained the most detected marker [19]. Carbapenems are the drug of choice for the treatment of infections caused by ESBL producers, but an increasing trend of resistance against these drugs has been reported in many Asian countries, including Pakistan [20]. The problem of emerging resistance in CRE along with poor prognosis in developing countries has resulted in a higher mortality rate of up to 40% [21]. The production of carbapenemases such as KPC, VIM, IPM, and NDM is a common feature in CRE isolates. *bla*_NDM_ is the most prevalent marker in Asian countries [20], followed by *bla*_KPC_, *bla*_IMP_, *bla*_VIM_, and *bla*_OXA-48_ [21,22].

Cefiderocol (CFDC) and eravacycline (ERV) have been introduced lately as options against carbapenem-resistant (CR) isolates with an efficacy of 70% to 95% against MBL producers [23]. However, these drugs are not available in many developing countries, and hence the data related to resistance against these drugs has not been widely reported. It is also worthy to note that the prevalence of genetic variants of ESBLs and carbapenemases in Pakistan has not been studied extensively, particularly in uropathogens isolated from diabetic patients. Keeping this lag in view, the current study was aimed at understanding the basis of antibiotic resistance among Enterobacteriaceae uropathogens; the data will also help to improve antibiotic surveillance and stewardship guidelines.

## 2. Materials and Methods

### 2.1. Retrieval of Data, Bacterial Isolates, and Identification

The data related to the samples was obtained from several laboratories across the province of Khyber Pakhtunkhwa, Pakistan during the study period from January 2018 to December 2018. The data related to the patients’ age and sex, signs and symptoms of UTI, recurrence of UTI, duration, and type of diabetes was collected. Comorbidity other than diabetes, taking antibiotics, and refusal to provide consent were taken as exclusion criteria. In the case that the same patient appeared twice but with a different pathogen species or resistance type, it was considered as a new sample. Isolates other than Enterobacteriaceae were not included in this study. All the patients of UTI with diabetes of both sexes and without discrimination of age were included in this study. The laboratory data of 910 uropathogens was assessed to select Enterobacteriaceae isolates which belonged to *E. coli, K. pneumoniae, P. mirabilis,* and *E. cloacae.* The sample processing and bacterial isolation were carried out at the collaborating laboratories. Briefly, urine was inoculated on a cystine–lactose–electrolyte-deficient (CLED) medium and incubated at 37 °C for 24–48 h. The initial identification of the isolates was carried out by traditional methods, including Gram-staining, cultural characteristics, and biochemical tests (such as citrate utilization, oxidase, urea and indole test, and TSI). The identification was confirmed by API 20E (Biomerieux, Lyon, France).

### 2.2. Antimicrobial Susceptibility Testing

To investigate antibiotic sensitivity, the isolates were grown on MacConkey agar plates for 24 h, and an inoculum standardized with 0.5 McFarland was transferred onto Mueller–Hinton agar plates to prepare a uniform bacterial lawn. Antibiotic discs were placed at appropriate distances. For antimicrobial sensitivity, the panel of antibiotics included amoxicillin/clavulanic acid; aztreonam; trimethoprim-sulphamethoxazole; cefepime; cefoxitin; ceftazidime; tetracycline: levofloxacin; gentamicin; nitrofurantoin; piperacillin/tazobactam; norfloxacin; ciprofloxacin; meropenem; imipenem; ampicillin; cefazolin; fosfomycin; amikacin; ceftriaxone; ertapenem; and doripenem. The isolates were categorized as sensitive, intermediate, or resistant according to the CLSI (2017) guidelines.

Susceptibility to colistin was performed by the colistin broth disk elution method following the protocol given in the CLSI (2021) guidelines. Briefly, Mueller–Hinton broth (MHB) with cation adjusted was arranged in four sets where each tube contained 10 mL MHB. The tubes were labeled as 0, 1, 2, and 4. One tube was kept as control (0), while in the remaining tubes, 1, 2, and 4 colistin discs (10 µg; OXOID, Basingstoke, UK) were added to obtain 0, 1, 2, and 4 µg mL^−1^ of the final concentration of colistin in the respective tubes. Colistin was eluted by vortexing and by keeping the tubes at room temperature for half an hour, following which the inoculum was added and the turbidity was adjusted to the 0.5 McFarland standard. The tubes were incubated at 37 °C for 24 h. The growth was inspected visually, and the minimum inhibitory concentration (MIC) was determined. The isolates were categorized as intermediate or resistant according to the CLSI guidelines.

In addition to routinely prescribed combinations, novel regimens of cefiderocol (FDC 30 µg; Liofilchem srl, Italy) and eravacycline (ERV 20 µg; Liofilchem, Italy) were also tested for antibacterial sensitivity by following the guidelines given by the Clinical and Laboratory Standards Institute and the Food and Drug Administration (FDA). The breakpoint criteria of ≤11 and ≤15 mm was used for FDC and ERV, respectively.

### 2.3. Phenotypic Characterization of ESBLs

The isolates having resistance to one or more cephalosporin or aztreonam were further tested for ESBL production through a double disc synergy test as described previously in the CLSI (2017) guidelines. The isolates were grown at 37 °C for 20 h on MHA plates containing discs of cephalosporin, aztreonam, and amoxicillin (with clavulanic acid). The zone of inhibition around cephalosporin or aztreonam was noted for any extension towards the other combination, and they were then categorized as ESBL producers.

### 2.4. Phenotypic Characterization of Carbapenemases

Initially, carbapenem resistance was assessed by the Kirby–Bauer disc diffusion method using MHA. The isolates were spread over the plates to make lawns, and four carbapenem discs (10 µg, each), including imipenem, meropenem, ertapenem, and doripenem were placed at appropriate distances. The plates were incubated at 37 °C for 20 h and the results were interpreted as per the CLSI guidelines.

The isolates exhibiting resistance to carbapenem were phenotypically assayed using EDTA and phenylboronic acid for MBL and KPC production. The cultures were inoculated on MHA by making lawns. Imipenem (IMP, 10 µg) and meropenem (MEM, 10 µg) discs were placed in three different sets; besides one set as control, the other two sets were either added with 0.5 mg of EDTA or 400 mg of phenylboronic acid. After cultivation at 37 °C for 20 h, an increase in the zone of inhibition (5–7 mm) around EDTA- or phenylboronic-acid-impregnated discs was compared with the control, and such isolates were marked as MBL/KPC producers.

### 2.5. Genotypic Characterization of ESBLs and Carbapenemases

Phenotypically screened ESBLs and carbapenemase producers were genotypically analyzed by two methods, including manual PCR and Check-MDR CT103XL microarray. The Check-MDR CT103XL microarray kit (15LTN0384) was used following the manufacturer’s instructions. DNA was extracted by the kit method (WizPrep^TM^ gDNA Mini Kit, Seongnam-si, Korea). The relevant genes of the ESBLs and carbapenemases were also determined by manual PCR using the primers and conditions mentioned in the Table 1. The results of manual PCR were compared with microarray analysis.

### 2.6. Statistical Analysis

SPSS ver 26.0 was used to analyze the data and most of the values are given as frequencies and their percentages.

## 3. Results

### 3.1. Clinical and Other Variables

Considering the sharp increase in diabetes and related complications, the health sectors in developing countries are over-burdened. Repeated occurrence of UTIs caused by antibiotic-resistant bacteria is common in diabetic patients. This study was focused on the antibiotic-resistance characteristics of Enterobacteriaceae uropathogens isolated from diabetic patients. Table 2 indicates that most of the patients were suffering from type 2 diabetes with very poor control of the blood glucose level. Fasting blood sugar level, at least once during the active infection, exceeding 120 mg dL^−1^ was taken as a measure of control along with HbA1c > 6.5% [30]. Most of the patients were female, with a large proportion of them being middle aged (33.81%) to elderly women (50.64%), indicating the relationship between the reproductive stage of women with susceptibility to UTIs. A total of 910 isolates were collected from the 879 patients; 31 isolates obtained from previously included patients but were different either in their resistance profile or belonging to a different species. Such cases were considered as recurrent UTI. The samples showing the two or three pathogens including Gram-negative rods other than the required species and Gram-positive bacteria were not considered here. Only 209 isolates were from male patients. More than 90% of the patients had diabetes for a duration of more than a year and even for more than a decade.

### 3.2. Prevalence of Major Uropathogens and Antimicrobial Susceptibility Pattern

Amongst Enterobacteriaceae, *E. coli* was the most frequently isolated pathogen (Table 3) followed by *K. pneumoniae*. While *Enterobacter cloacae* and *P. mirabilis* appeared less frequently.

The antimicrobial susceptibility testing presented a high level of resistance amongst the isolates, which can be a reason for the repeated episodes of UTI in diabetic patients. Piperacillin-tazobactam and tetracycline were found to be the most ineffective drugs with a resistance level of >80% (Table 4). The resistance to cephalosporins in *E. coli* was also alarmingly high (56–75%). More than two-thirds of the isolates showed resistance to amoxicillin-clavulanic acid, aztreonam, levofloxacin, and norfloxacin. The resistance to carbapenems was, however, lower (247; 27.14%) than the other antibiotics.

### 3.3. Resistance to Colistin

Although the isolates did not exhibit a higher rate of resistance towards colistin compared to the other tested antibiotics (Table 5), the rate was more than reported earlier for *E. coli* and *K. pneumoniae.* It is also important to note that colistin is still not a routinely prescribed medicine to patients in this part of the world. However, its use in the veterinary sector has been stated by the concerned persons. Nonetheless, the emergence of multi-drug resistant bacteria necessitates that caution be taken in prescribing this antibiotic.

### 3.4. Phenotypic Resistance Categories

The isolates were categorized as multidrug-resistant (MDR), extensively drug-resistant (XDR), and pan-drug resistant (PDR) by following guidelines given in Magiorakos et al. (2012) [31]. Out of 910 isolates, 421 (46.26%), 239 (26.26%), and 101 (11.09%) were found to be MDR, XDR, and PDR, respectively.

### 3.5. Phenotypic and Genotypic Characterization of Resistance Markers

In an attempt to characterize ESBL production by these uropathogens, phenotypic and genotypic assays were used. The isolates showing resistance to 3rd and 4th generation cephalosporin and/or aztreonam were considered as ESBL producers and were selected for phenotypic and genotypic analysis. Out of 910 isolates, 670 (73.62%) were resistant to 3rd and 4th generation cephalosporin. The double disc diffusion method presented 495 (54.39%) isolates as ESBL-positive. These phenotypically positive isolates were confirmed for the ESBL type by the Microarray technique. ESBL-PER was the most common in these isolates, except for *P. mirabilis* isolates which showed a lower rate (8.33%) for this marker. The data from the Microarray was affirmed by the manual PCR for ESBL types; however, the sensitivity of the PCR was lower than the Microarray (Table 6). Nonetheless, the pattern of ESBL types did not vary to a greater extent from the data that has been stated earlier for this geographic region, emphasizing the widespread transfer of the resistance genes among pathogens.

MBL production appeared as the most widely distributed feature in CR isolates (Table 7). Arguably, the level of MBL (65.71%) was even higher in *K. pneumoniae* isolates than the level of KPC (30.95%). The study indicated the endemic nature of MBL, particularly among CRE uropathogens. This finding can definitely aid physicians in prescribing antibiotics. The data from genotypic characterization revealed *bla*_NDM_ as the predominant marker followed by the *bla*_KPC_ encoding gene. *bla*_IPM_ and *bla*_VIM_ could not be detected in any CR isolate. The mechanism of resistance in a few isolates could not be recognized here; however, it can be attributed to the presence of chromosomal AmpCs, efflux pump, or change in permeability or target site. The results of microarray and manual PCR were almost similar except for the NDM in *E. coli*. Repeated investigations using manual PCR affirmed this finding.

### 3.6. Co-Existence of Resistance-Encoding Genes

**Figure 1:**
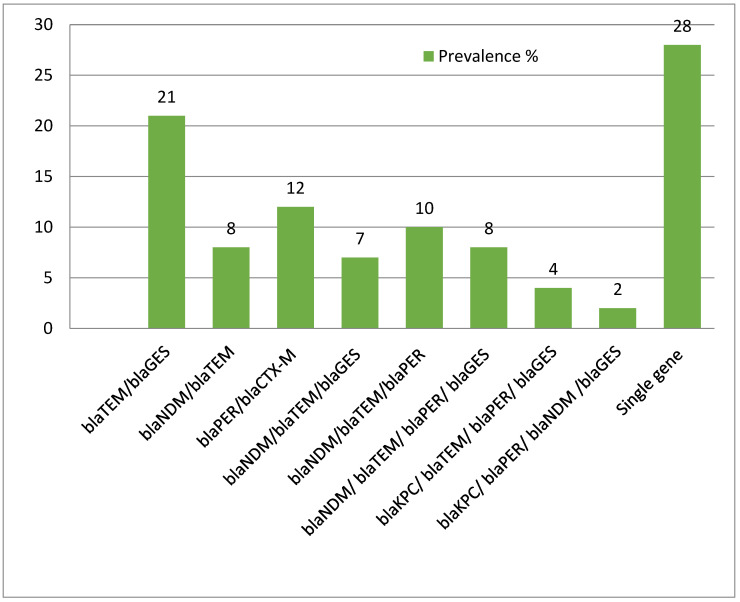
Prevalence of co-existing resistance genes in isolates. (y-axis represents % of the isolates).

Out of 495, most of the isolates exhibited a co-existence of resistance genes (Figure 1). Indeed, the antimicrobial resistance spectrum was significantly increased with the co-existing genes (*p* < 0.01). *bla*_TEM_ and *bla*_GES_ were the most frequent pair of resistance markers in these isolates.

### 3.7. Resistance to Cefiderocol and Eravacycline

The resistance to new regimens, cefiderocol and eravacycline, was conceivably lower except for *P. mirabilis* which was completely resistant to eravacycline (Table 8). These regimens are not available in developing countries and hence it was presumed that these drugs will exhibit good sensitivity. However, the presence of complete resistance in *P*. *mirabilis* necessitates the assessment of the effectiveness of the new regimen in the near future, particularly with larger datasets.

## 4. Discussion

Diabetes mellitus has been recognized as a predisposing factor for UTIs [32,33]. The incidence of UTIs in women is higher and can cause more complications if the infection reaches the kidney [8]. The findings in the current study also corroborate that women, particularly those suffering from type 2 diabetes, are more susceptible to UTIs. It is generally believed that sexually active women are less susceptible to UTIs [34], which was also supported by the current findings as a large number of patients were older women.

*E. coli* has been described as a leading cause of UTI among all the bacteria [35] and the most common uropathogen in diabetic patients [32,36]. The findings of this study, despite being skewed towards Enterobacteriaceae, also corroborated it. The involvement of Enterobacteriaceae isolates in UTIs has widely been reported [37]; however, the data specifically obtained from diabetic patients is scarcely available. Antimicrobial resistance is a global issue and a challenge for clinicians because of the rapid increase in resistance. In the present study, the majority of the isolates were found to be resistant to commonly used antibiotics, including ampicillin, cefazolin, ceftriaxone, and amoxicillin-clavulanic acid. A considerable proportion was found to be MDR, while XDR and PDR were less common. The prevalence of these drug-resistant categories reportedly varies in different parts of the world. Pakistan, being a South Asian developing country, contributes a significant burden in AMR [38], with a high level (63–100%) of MDR and XDR bacteria [38]. Abbas et al. (2020) [39] reported a higher level of XDR (56%) strains of *E. coli* and *Klebsiella* species than the current findings. The resistance to colistin in Enterobacteriaceae is previously not reported from Peshawar, Pakistan [40]. However, in the present study, high values of MICs were obtained for the isolates from this region

In Pakistan, ESBLs have widely been reported in MDR bacterial strains with an overall frequency of 40% [41]. Here, it appeared higher (54.3%) in the isolates from diabetic patients. It can be linked with the presence of resistant bacteria in such patients, particularly isolates from the Enterobacteriaceae family. Abrar et al. (2018) [41] performed a meta-analysis where CTX-M appeared as a common ESBL variant in this region. The other variants reported from Pakistan included SHV and TEM. Here, PER appeared as a dominant marker. Previously, PER-1-type ESBLs have been reported in the *P. aeruginosa* and *Acinetobacter* species from Pakistan [42]. The current finding needs more investigation to ascertain this change in genotypic marker. Nonetheless, the commonly found ESBL genes, including CTX-M, GES, and TEM [7,43], also appeared in this study. The current findings regarding the absence of SHV confirmed a previous report from this region [44].

Antibiotic resistance is a global threat especially in immunocompromised or susceptible individuals such as chronic diabetic patients. The data showed that the resistance to reserve or less-prescribed drugs, including carbapenems and colistin, is higher [20]. Indeed, the resistance to carbapenems was higher as compared to a previous study from Karachi, Pakistan [45]. CREs have also been linked with high mortality and morbidity. Likewise, the resistance to colistin has also been increased compared to an earlier finding [20]. Colistin is considered as a last resort for the treatment of CR bacteria. Various factors are associated with the increased resistance to antibiotics, including over-the-counter sale, the irrational use of antibiotics, and lack of antibiotic stewardship programs. Enterobacteriaceae isolates, notably *E. coli*, *K. pneumoniae* and *E. cloacae,* harbor resistance to a variety of antibiotics, particularly by elaborating β-lactamases [20]. The incidence of as high as 32% carbapenem resistance has also been reported in these isolates, leading to an expansion of the list of PDR bacteria [46].

CRE commonly and frequently produce KPC and NDM as modes of action against antibiotics [20]. In this study, the resistance to β-lactam antibiotics was found higher, which could be attributed to the production of ESBLs and modification in surface receptors [47]. Among carbapenemase producers, NDM was the prevalent marker that confirmed an earlier finding from the same geographic region [20]. Habib et al. (2022) [48] also reported the widespread nature of NDM in CRE isolates from this region. Here, the majority of cases of CRE were of carbapenemase-producing Enterobacteriaceae (CPE), as reported in a recent report [48].

Lately, the survey of data available regarding eravacycline showed a cumulative (%) degree of MIC for non-MDR Enterobacteriaceae in the range of 0.5–1 µg mL^−1^, while for MDR isolates, it was 2–8 µg mL^−1^ [49]. The data was particularly encouraging as this fluorocycline drug was developed to treat infections caused by MDR bugs [50]. The drug is not available in this part of the world; however, the resistance pattern indicated the extent of the global antimicrobial resistance pattern, and therefore, the introduction of new drugs in any region should be based on a larger dataset (that should also be reviewed periodically). Considering this lag in view, a study published from China in 2022 investigated the efficacy of cefiderocol against CRE isolates [51], and deciphered the role of cirA, pbp3, and *bla*_NDM-5_ in the resistance to this drug. The siderophore-related gene cirA was of particular interest as cefiderocol is a catecholamine-siderophore-derived cephalosporin [52]. This study affirmed the hypothesis that even before the introduction of a drug, resistance can be found in the local isolates as resistance against cefiderocol and eravacycline was found in ~8 and 15% of the isolates, respectively. The isolates with the NDM and PER combination exhibited resistance to cefiderocol as previously reported [53].

## 5. Conclusions

Urinary tract infections are common amongst patients of type 2 diabetes. The uropathogens, particularly the members of Enterobacteriaceae, exhibit a high level of drug resistance. Antibiotic resistance relies on various mechanisms, including the production of extended spectrum β-lactamases (ESBLs) and carbapenemases. The isolates investigated in this study exhibited PER and TEM variants as the prevalent ESBLs. The presence of high levels of PER needs further investigation as it has not been reported earlier from this geographic region. Among carbapenem-resistant isolates, *bla*_NDM_ was the common mode of resistance; a large proportion of such isolates showed the co-existence of β-lactamases with carbapenemases. The mechanisms of resistance other than ESBLs and carbapenemases need to be explored in future studies. Colistin along with relatively new drugs, cefiderocol and eravacycline, appeared effective, and hence can be regarded as the drugs of choice for the treatment of UTIs in diabetic patients. This study provides a basis for improving antibiotic surveillance and stewardship programs.

## Figures and Tables

**Table 1:** Primers and details for the amplification of common ESBLs and carbapenemases.

Primer	Sequence	Annealing Temperature °C	Product Size bp	Reference
TEM	F-ATGAGTATTCAACATTTCCG R-TTAATCAGTGAGGCACCTAT	51	851	Grimm et al., 2004 [24]
SHV	F-GCAAAACGCCGGGTTATTC R-GGTTAGCGTTGCCAGTGCT	57.4	940	Gröbner at el., 2009 [25]
PER	F-GCTCCGATAATGAAAGCGT R-TTCGGCTTGACTCGGCTGA	53	520	Uddin et al., 2022 [20]
GES	F-GTT AGA CGG GCG TAC AAA GAT AAT R-TGT CCG TGC TCA GGA TGA GT	55	903	Ryoo et al., 2005 [26]
CTX-M	F-CGCTTTGCGATGTGCAG R-ACCGCGATATCGTTGGT	55.7	551	Paterson et al., 2003 [27]
NDM	F-CACCTCATGTTTGAATTCGCC R-CTCTGT CACATCGAAATCGC	60	984 bp	Kaase et al., 2011 [28]
KPC	F-CAGCTCATTCAAGGGCTTTC R-AGTCATTTGCCGTGCCATAC	56	533	Gröbner at el., 2009 [25]
VIM	F-GGTGTTTGGTCGCATATCGC CCATTCAGCCAGATCGGCATC	65	503 bp	Wolter et al., 2009 [29]
IMP	F-GGAATAGAGTGGCTTAATTC R-CAACCAGTTTTG CCTTACC	53	327 bp	Wolter et al., 2009 [29]

**Table 2:** Status of diabetes as informed by the patients (*n* = 910).

Name of Variable	Occurrence
Type of DM	
Type 1	264 (29.02%)
Type 2	646 (70.98%)
Gender	
Male	209 (22.96%)
Age (years)	
1–20	1 (0.47%)
20–40	32 (15.31%)
40–60	107 (51.19%)
60–80	61 (29.18%)
>80	8 (3.82%)
Female	701 (77.03%)
Age (years)	
1–20	3 (0.42%)
20–40	49 (6.99%)
40–60	237 (33.81%)
60–80	355 (50.64%)
>80	57 (8.13%)
Blood glucose level	
Fasting Blood Sugar *	
≥120	115 (12.63%)
<120	795 (87.36%)
HbA1c	
<6.5%)	293 (32.2%)
>6.5%	617 (67.8%)
Duration of DM	
<1 year	72 (7.91%)
1–10 years	420 (46.15%)
>10 years	418 (45.94%)

* The data was obtained during the course of infection at least once per patient.

**Table 3:** Prevalence of enteric uropathogens from diabetic patients (*n* = 910).

Pathogen	Prevalence
*Escherichia coli*	536 (58.90%)
*Klebsiella pneumoniae*	337 (37.03%)
*Enterobacter cloacae*	19 (2.08%)
*Proteus mirabilis*	18 (1.97%)

**Table 4:** Antimicrobial resistance patterns of Enterobacteriaceae uropathogens.

Antibiotics	*E. coli* (*n* = 536)	*K. pneumoniae* (*n* = 337)	*Enterobacter cloacae* (*n* = 19)	*P. mirabilis* (*n* = 18)	Total (*n* = 910)
AMC	458 (85.44%)	208 (61.72%)	18 (94.73%)	16 (88.89%)	700 (76.91%)
ATM	382 (71.26%)	203 (60.23%)	16 (84.21%)	15 (83.33%)	616 (67.69%)
SXT	324 (60.44%)	241 (71.51%)	11 (57.89%)	12 (66.67%)	588 (64.61%)
FEP	402 (75.00%)	210 (62.31%)	13 (68.82%)	11 (61.11%)	636 (69.89%)
FOX	301 (56.16%)	177 (52.52%)	19 (100%)	10 (55.55%)	507 (55.71%)
CAZ	404 (75.37%)	209 (62.01%)	13 (68.82%)	12 (66.67%)	638 (70.10%)
TE	415 (77.42%)	283 (83.97%)	16 (84.21%)	18 (100%)	732 (80.43%)
LEV	406 (75.74%)	239 (70.91%)	16 (84.21%)	14 (77.78%)	675 (74.17%)
GN	301 (56.16%)	216 (64.09%)	17 (89.47%)	14 (77.78%)	548 (60.21%)
F	321 (59.88%)	183 (54.30%)	15 (78.94%)	18 (100%)	537 (59.01%)
TZP	441 (82.27%)	204 (60.53%)	15 (78.94%)	14 (77.78%)	674 (74,06%)
NOR	410 (76.49%)	255 (75.66%)	14 (73.68%)	11 (61.11%)	690 (75.82%)
CIP	410 (76.49%)	255 (75.66%)	11 (57.89%)	12 (66.67%)	688 (75.60%)
MER	138 (25.74%)	91 (27.01%)	04 (21.05%)	05 (27.78%)	238 (26.15%)
IMP	142 (26.49%)	88 (26.11%)	06 (31.57%)	05 (27.78%)	241 (26.45%)
AMP	465 (86.75%)	263 (78.04%)	19 (100%)	16 (88.89%)	763 (83.84%)
CFZ	462 (86.19%)	260 (77.15%)	19 (100%)	15 (83.33%)	756 (83.07%)
FOS	181 (33.76%)	111 (32.93%)	05 (26.31%)	06 (33.33%)	303 (33.29%)
AK	176 (32.83%)	177 (52.52%)	13 (68.82%)	10 (55.55%)	376 (41.31)
CRO	406 (75.74%)	230 (68.24%)	19 (100%)	15 (83.33%)	670 (73.62%)
ERT	142 (26.49%)	88 (26.11%)	06 (31.57%)	08 (44.44%)	244 (26.81%)
DOR	142 (26.49%)	88 (26.11%)	07 (36.84%)	10 (55.55%)	247 (27.14%)

Abbreviations: AMC, amoxicillin/clavulanic acid; ATM, aztreonam; SXT, trimethoprim-sulphamethoxazole; FEP, cefepime; FOX, cefoxitin; CAZ, ceftazidime; TE, tetracycline: LEV, levofloxacin; GN, gentamicin; F, nitrofurantoin; TZP, piperacillin/tazobactam; NOR, norfloxacin; CIP, ciprofloxacin; MER, meropenem; IMP, imipenem; AMP, ampicillin; CFZ, cefazolin; FOS, fosfomycin; AK, amikacin; CRO, ceftriaxone; ERT, ertapenem; DOR, doripenem.

**Table 5:** Resistance of the Enterobacteriaceae uropathogen against colistin. The isolates with MIC ≤ 2μg/ mL were considered as intermediate, and isolates with ≥4 µg/mL were taken as resistant. The criteria for sensitivity is not mentioned in CLSI (2021).

Bacterial Isolate	Intermediate (≤2 µg/ mL)	Resistant (≥4 µg/mL)	Total *n* (%)
*E. coli* (*n* = 536)	489 (91.24%)	47 (8.76%)	536(100)
*K. pneumoniae* (*n* = 337)	302 (89.62%)	35 (10.38%)	337(100)
*Enterobacter cloacae* (*n* = 19)	18 (94.74%)	1 (5.26%)	19(100)
*P. mirabilis* (*n* = 18)	00	18 (100%)	18(100)
Total (*n* = 910)	809 (88.90%)	101 (11.10%)	910(100)

**Table 6:** Detection of ESBL production using phenotypic method, manual PCR, and microarray assay.

Isolates	Phenotypic Detection	Manual PCR (*n* = 495) (%)					Microarray Assay (*n* = 495)				
		TEM	PER	GES	CTX-M	Unrecognized	TEM	PER	GES	CTX-M	Unrecognized
*E. coli*	312 (58.2)	83 (26.6)	134 (42.9)	34 (10.8)	39 (12.5)	22(7.0)	85 (27.2)	139 (44.5)	36 (11.5)	41 (13.14)	11 (3.52)
*K. pneumoniae*	165 (48.9)	55 (33.3)	79 (47.8)	5 (3.0)	13 (7.8)	13(7.8)	59 (35.7)	81 (49)	6 (3.6)	14 (8.48)	05 (3.0)
*Enterobacter cloacae*	08 (42.1)	02 (25)	02 (25)	00	3 (37.5)	1 (12.5)	02 (25)	02 (25)	0	04 (50)	Nil
*P. mirabilis*	10 (55.5)	02 (20)	02 (20)	02 (20)	02 (20)	Nil	04 (40)	02 (20)	02 (20)	02 (20)	Nil
Total *n* (%)	495 (54.3)	134 (27)	207 (41.8)	36 (7.2)	53 (10.7)	36(7.2)	150 (30.3)	224 (45.2)	44 (8.8)	61 (12.32)	16 (3.23)

**Table 7:** Characterization of carbapenemases into MBL and KPC on phenotypic basis and detection of carbapenemases encoding genes (*n* = 250).

Isolates	Phenotypic Methods	Total (%)	Manual PCR			Microarray Assay		
			*bla*_NDM_	*bla*_KPC_	Unrecognized	*bla*_NDM_	*bla*_KPC_	Unrecognized
*E. coli* (*n* = 142)	MBL	107 (75.35%)	109 (76.7%)	24 (16.90%)	9 (6.33%)	111 (78.16%)	24 (16.90%)	7 (4.92%)
	KPC	21 (14.78%)						
	Unrecognized	14 (8.85%)						
*K. pneumoniae* (*n* = 91)	MBL	61 (67.03%)	64 (70.3%)	27 (29.67%)	00	64 (70.3%)	27 (29.67%)	0
	KPC	27 (29.67%)						
	Unrecognized	3 (3.29%)						
*E. cloacae* (*n* = 7)	MBL	5 (71.42%)	5 (71.42%)	2 (28.58%)	00	5 (71.42%)	2 (28.58%)	0
	KPC	2 (28.58%)						
	Unrecognized	-						
*P. mirabilis* (*n* = 10)	MBL	5 (50.0%)	5 (50.0%)	5 (50%)	2 (20%)	5 (50%)	5 (50%)	0
	KPC	3 (30.0%)						
	Unrecognized	2 (20%)						
Total (*n* = 250)	MBL	178 (71.2%)	183 (73.2%)	55 (22%)	11 (4.4%)	188 (75.2%)	55 (22%)	7 (2.8%)
	KPC	53 (21.2%)						
	Unrecognized	19 (7.6%)						

**Table 8:** Resistance to a new regimen, cefiderocol (FDC) and eravacycline (ERV), in CRE isolates (*n* = 250).

Isolate	Resistant to	
	FDC	ERV
*E. coli* (*n* = 142)	10 (7.04)	19 (13.38)
*K. pneumoniae* (*n* = 91)	7 (7.69)	9 (9.89)
*Enterobacter cloacae* (*n* = 7)	2 (28.57)	1 (14.28)
*P. mirabilis* (*n* = 10)	1 (10.0)	10 (100)
Total (*n* = 250)	20 (8.0)	39 (15.6)

## Data Availability

Data associated with this manuscript can be obtained from the corresponding author upon reasonable request.
